# Community-Led Solutions to Address Black Maternal and Infant Mortality Through the TNT-PISP Model: A Qualitative Study

**DOI:** 10.1007/s10995-026-04264-1

**Published:** 2026-05-02

**Authors:** Morgan S. White, Deejay Zwaga, Laura E. T. Swan, Baillie Frizell-Thomas, Obiageli Oniah, Jasmine Y. Zapata

**Affiliations:** 1 Department of Family Medicine and Community Health, School of Medicine and Public Health, University of Wisconsin- Madison, Madison, USA; 2 School of Medicine and Public Health, University of Wisconsin-Madison, Madison, USA; 3 Reproductive Equity Action Lab, School of Medicine and Public Health, University of Wisconsin-Madison, Madison, USA; 4 Department of Family Medicine, University of Colorado, Boulder, USA; 5 Department of Pediatrics, School of Medicine and Public Health, Department of Health Services, University of Wisconsin-Madison & Wisconsin, Madison, USA

**Keywords:** Community health, Health disparities, Prenatal care, Support groups

## Abstract

**Objectives:**

In both the U.S. and Wisconsin, Black women and infants experience significantly higher rates of morbidity and mortality than their white counterparts. Our research team set out to explore how a community-based and culturally informed perinatal support model could address the needs of Black mothers and their families.

**Methods:**

We developed and implemented the Today Not Tomorrow Pregnancy and Infant Support Program (TNT-PISP), a community-based, culturally informed perinatal support model integrated with traditional obstetrical care. From October 2019 to August 2022, we held monthly support group sessions facilitated by Black community-based doulas, Black physicians, and community partners. Twenty-five participants engaged in topic-focused and freeform sessions to discuss mental health, breastfeeding, peripartum care, and medical racism. Data were collected through semi-structured interviews and focus groups and analyzed using the Daughtering Method and reflexive thematic analysis.

**Results:**

Participants emphasized the importance of shared Black identity and culture in fostering connection and trust. The group’s open, judgment-free environment allowed for meaningful conversations and emotional support. Participants valued the exchange of parenting knowledge and community resources. The program’s flexible structure and child-friendly setting were key strengths, enabling consistent participation despite busy schedules.

**Conclusions for Practice:**

This study highlights the potential of community-based, culturally informed perinatal support programs to promote health equity for Black women and infants. Future research should explore such programs’ long-term impacts and scalability in diverse settings. Continued efforts to integrate culturally relevant care models into traditional healthcare systems may help promote health inequities in Black communities.

**Supplementary Information:**

The online version contains supplementary material available at 10.1007/s10995-026-04264-1.

## Significance

There is a growing body of evidence that suggests that group prenatal care, doula care, culturally centered care models, and community-based peer support groups may play a role in reducing Black maternal and child healthcare disparities. Our qualitative study explores how Black participants engage with a community-based pregnancy peer support group and what participants viewed as support group strengths. Our study provides evidence that a pregnancy support group may be one setting where healthcare providers and public health professionals can promote interventions to address healthcare disparities.

**Supplementary Information:**

The online version contains supplementary material available at 10.1007/s10995-026-04264-1.

## Background

Black women and infants in the United States face disproportionately high rates of morbidity and mortality compared to their white counterparts (Ely & Driscoll, 2024; Howell et al., 2016; Hoyert, 2024; K. L. Liese et al., 2019). In Wisconsin, the data show a similar pattern: Black women and infants experience the highest morbidity and mortality rates (Wisconsin Department of Health Services, 2022, 2024b; Wisconsin Department of Health Services, Division of Public Health, Office of Health Informatics, n.d.). Specifically, non-Hispanic Black women in Wisconsin are 2.5 times more likely to die during or shortly after pregnancy compared to non-Hispanic white women (Wisconsin Department of Health Services, 2022, 2024b). Similarly, infants born to non-Hispanic Black mothers in Wisconsin are three times more likely to die than infants born to non-Hispanic white mothers (Wisconsin Department of Health Services, Division of Public Health, Office of Health Informatics, n.d.). Pre-term birth and low birth weight are major drivers of Black infant deaths in Wisconsin (Wisconsin Department of Health Services, 2023), and both of these outcomes for Black infants have been associated with structural racism (Britton & Shin, 2013; Debbink & Bader, 2011; Geronimus, 1996; Mason et al., 2009; Wallace et al., 2015). In addition, income inequality, residential segregation, and chronic stress have all been proposed as possible structural factors contributing to low birth weight in Black infants (Britton & Shin, 2013; Debbink & Bader, 2011; Geronimus, 1996; Mason et al., 2009; Wallace et al., 2015).

Against this backdrop of persistent inequities, findings from a community engagement report conducted in our local Wisconsin community revealed that Black women identified limited social support and the lack of culturally relevant models of care as barriers to optimal quality healthcare and contributors to poor birth outcomes (The Foundation for Black Women’s Wellness & EQT by Design, 2019). While evidence suggests that group prenatal care (Liese et al., 2022; *The JJ Way® - Commonsense Childbirth Inc.*, 2022), doula care (Kozhimannil et al., 2013; Lemon et al., 2024; Ramey-Collier et al., 2023), culturally centered care models (Hardeman et al., 2020), and community-based peer support groups may play a role in reducing Black maternal and child healthcare disparities (Duncan et al., 2022; Shakya et al., 2017; Ware et al., 2021), few studies have designed interventions explicitly grounded in community-identified needs within the local context of persistent disparities. Despite ongoing efforts across policy, healthcare, and community sectors, disparities in Black maternal and infant outcomes have persisted in Wisconsin (Wisconsin Department of Health Services, 2024b, 2024a).

To address this gap, our research team collaborated with community partners to develop and implement the Today Not Tomorrow Pregnancy and Infant Support Program (TNT-PISP), a community-based, culturally informed perinatal support model intentionally designed in response to the barriers identified in the community engagement report. This grounding in community-derived priorities represents a novel contribution to the literature, offering an intervention co-developed with Black women to address locally identified shortcomings in social support and culturally relevant care. Our study adds to the growing body of literature indicating that Black women engage positively with culturally relevant care models and provides new evidence on how such models can be integrated with traditional obstetrical care to better meet the needs of Black women and their families.

## Methods

### Today Not Tomorrow Pregnancy and Infant Support Program

**Fig. 1:**
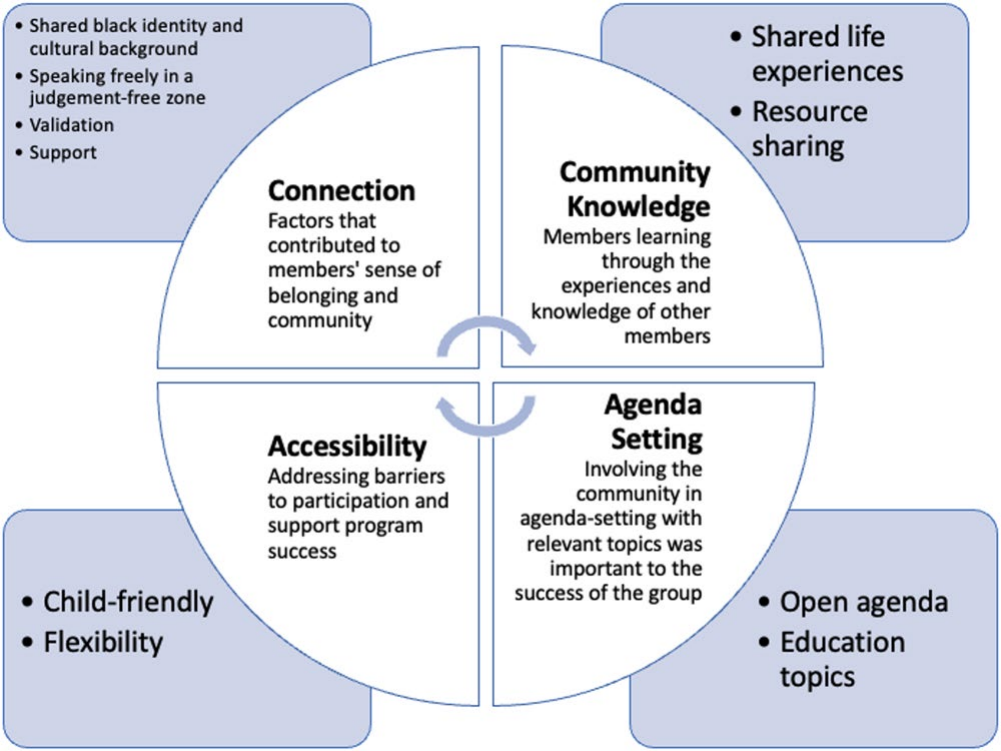
The qualitative themes identified through our reflexive thematic analysis, with bulleted sections identifying major ideas described in each theme

Our female research team collaborated with community partners and an interprofessional team of healthcare providers to develop a program that integrated three evidence-based models: (1) community-based doula programs (Kozhimannil et al., 2013; Lemon et al., 2024; Ramey-Collier et al., 2023), (2) group-based prenatal care (Liese et al., 2022; *The JJ Way® - Commonsense Childbirth Inc.*, 2022), and (3) community-based pregnancy support groups (Duncan et al., 2022; Shakya et al., 2017; Ware et al., 2021). Through partnerships with local community organizations serving Black families, we recruited self-identified Black women who were pregnant or had delivered within the past year in our local Wisconsin community. A total of 25 people consented to participate in the program. (Fig. 1

We conducted monthly support group sessions from October 2019 to August 2022. These sessions were facilitated by a multidisciplinary team, including Black community-based doulas, Black physicians, and community partners. Initially, the program featured two-hour in-person meetings; however, these were adapted to 90-minute virtual meetings in response to the coronavirus disease 19 (COVID-19) pandemic. In-person sessions were offered at a convenient community location familiar to participants. The meeting was child-friendly, and meals were provided for participants and their accompanying family members.

The principal investigator and a community-based doula selected support group topics based on feedback collected during qualitative one-on-one and group interviews the year before the launch. Topics included, but were not limited to, mental health, breastfeeding, peripartum care, healing from past birth trauma, support networks, and medical racism.

### Data Collection

We invited TNT-PISP participants to complete interviews during their prenatal period, early postpartum period, and approximately six months postpartum, aiming to conduct three individual interviews with each participant. Between October 2019 and March 2021, six participants completed one interview, three completed two interviews, and two completed three interviews, resulting in a total of 11 people who participated in at least one individual interview. We also conducted three virtual focus groups with a total of 14 unique participants. Focus groups 1 and 2 each had 7 unique participants; focus group 3 consisted of participants who had already participated in either an individual interview or in one of the first two focus groups. The first focus group occurred during the first month of the TNT-PISP in October 2019, the second occurred in December 2019, and the third focus group occurred in August 2022. Participants were asked to reflect on their pregnancy, birthing, life experiences, and engagement with TNT-PISP. The interview guide can be found in the supplemental appendix. Each interview and focus group lasted approximately 90 min and was facilitated by the principal investigator. Participants received $20 in compensation for each completed interview. All interviews were audio-recorded and transcribed verbatim with the participants’ consent. Medical students or a professional transcription service completed the transcription. Background information on the research team and participants can be found in the supplemental appendix. The Institutional Review Board at the University of Wisconsin-Madison approved all study procedures.

### Data Analysis

Venus Evans-Winters’ Daughtering Method inspired our data collection and analysis processes (Evans-Winters, 2019). This method aims to decolonize qualitative methods and center Black women’s experiences by grounding researchers in the historically socialized roles Black women have held and engaging the academic self through a culturally reflective lens. We also applied Braun and Clarke’s reflexive thematic analysis (Braun & and Clarke, 2006). A medical student and a family medicine resident independently double-coded each transcript under the supervision of a researcher trained in qualitative methods. The two coders collaborated with the research team to reconcile codes and identify four overarching themes related to Black birthing experiences. This process and these findings have been published elsewhere (Zapata et al., 2025). Subsequently, a second medical student and a family medicine attending independently reviewed the double-coded transcripts to identify themes specific to the support group aspect of the TNT-PISP. The research team met regularly to iteratively refine themes related to the participants’ experiences in the TNT-PISP support group and their suggestions for successful future programming. We have followed the COnsolidated criteria for REporting Qualitative research (COREQ) guidelines for reporting.

## Results

Four themes emerged describing participants’ experiences in the TNT-PISP, which may inform best practices for perinatal support programs for Black women: (1) connection through shared identity and social support, (2) community knowledge is valuable, (3) agenda setting for and by participants, and (4) accessibility is key (Fig. 1). Quotes with illustrative themes are in Table 1 of the supplemental appendix.

### Connection Through Shared Identity and Social Support

Participants discussed factors contributing to their sense of belonging in the support group and aspects that fostered a strong sense of community. Participants felt that a shared Black identity and cultural background were essential for the success of the support group, creating an environment in which participants felt like they could be themselves. One participant said:

I definitely want to see Black people [at the support group]. Not saying that white people, white women wouldn’t have an opinion or things like that, but I wouldn’t get why they’re really there unless they were a part of the study and just seeing how it compares. But in the support group, Black women [need to be there] so that we could be comfortable sharing what we want to share.

Many participants remarked on how this shared identity influenced their willingness to learn about pregnancy and motherhood from others. One participant noted, “And I think also that because every mother, every pregnancy is different, having access to so many Black women is essential because if anyone is gonna have a pregnancy similar to the one a mother is having…it would likely be a Black woman.”

Members appreciated speaking freely during group meetings without fear of judgment. One participant said, “Everyone got to tell their story. Everyone got to tell it with no judgment from anyone else.” Participants felt a deeper sense of connection as they were able to share their unfiltered selves. One participant described the authenticity she felt in the support group:

It was just really nice as—I’ve been using this term a lot—let your hair down kind of a thing. Whereby we can be our authentic selves because we don’t have enough spaces where we can be and to share. Even [though] what we say may not be the most correct, but things that are ailing us, bothering us, things we need clarity on.

This freedom to speak without fear of judgment created an environment where participants could build deep connections with one another in a way different than they typically experienced in other spaces.

The support group validated and normalized participants’ experiences. One participant shared, “it helps to be surrounded by a community of Black women going through similar experiences to help you feel validated and to help you feel that you know I’m not just making things up or over exaggerating. These are truly terrible experiences that I’m going through and it’s normal to feel this way.” This participant connected these feelings of validation to mental health, stating, “When you’re surrounded by people who don’t have those experiences, it’s easy for you to just be like, ‘Oh no, I should just dismiss this’ or ‘Maybe it’s just me.’ To have that community validation and support—I think that is really important for improving mental health.”

Although it was important for group members to acknowledge and discuss their shared experiences of medical racism, describing and hoping for positive perinatal experiences was also a key aspect of the support and validation experienced in the support group. Participants felt a strong sense of connection and described other members as part of their personal support systems. Participants consistently described the support group as a community. One participant described how the group was useful in “establishing community,” explaining how it helped with “sustaining these moms with wonderful other women, with children [of] various ages” and provided them with “support as they’re navigating that first year or two of life or [a] babe in utero.” As in-person community gatherings were limited during the COVID-19 pandemic, participants relied on the virtual community created by the support group to maintain social connections. One participant commented, “There’s so much support in that group. And you feel it. Even though it’s virtual, you feel it through the computer. You feel everyone caring about one another. And so, it’s just really special because I don’t feel—I’ve lived in [city name] for a while. And I’ve never had that kind of experience with other Black women.”

### Community Knowledge is Valuable

The value of community knowledge was highlighted when participants shared their experiences learning through others’ life experiences and about community resources at the support group. Participants especially appreciated hearing about others’ engagement with the healthcare system, motherhood, family dynamics, and breastfeeding. Participants indicated they felt more comfortable asking questions about day-to-day mothering with the support group rather than their healthcare providers. One participant highlighted this phenomenon, saying:

Yeah, I think for me just being able to learn like that from other moms and the wealth of knowledge shared and the different experiences and not…having to go talk to doctors or necessary medical people about just the day to day of being a mother and the different things that may come up that you can get answers from other mothers.

Another participant shared the importance of the participants being able to “talk through what their success and challenges are, what they’re doing in their pregnancy, you know, how they plan to care for their children afterwards.”

Participants shared community resources with one another, which was particularly important for those not from the area. Daycare was an appreciated topic of discussion, with one participant noting an experience she had during the support group, saying, “I just shared a concern about how expensive daycare is and people came from out [of] the woodwork like, ‘Oh, get on this list,’ or ‘try this place,’ or ‘this place had really great reviews.’” The support group was also a space where participants felt comfortable asking for more sensitive resources, such as mental health care, free diapers, and grocery support. One participant shared:

So I teach on campus and then my partner is a student on campus and so [another participant] was just saying you know y’all absolutely have access to these funds to kind of help kind of decrease your contribution to daycare expenses. And so if I had not had that conversation, I wouldn’t have even known to look that up. And so it felt like there were questions that I could ask these folks but could not have asked my colleagues at work or my HR representative at work or that they might not have even known the answer to.

### Agenda Setting for and by Participants

**Fig. 2:**
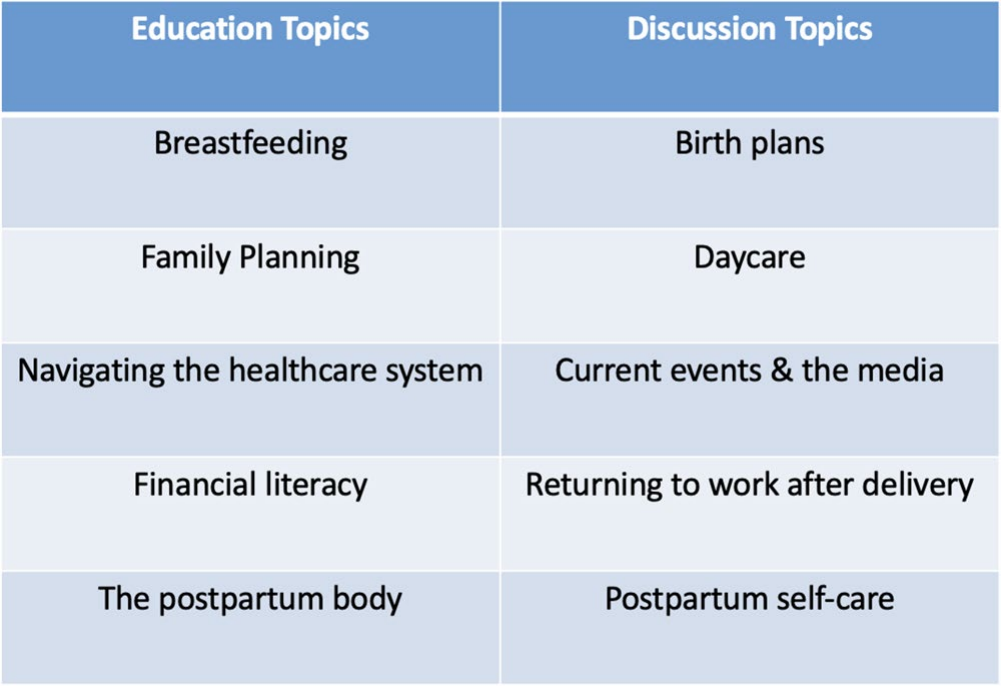
Participants’ suggested topics for future support groups, divided into topics that could be more educational (presented to the participants) vs. discussion-driven based on the personal experiences of participants

Scheduled education topics related to mothering were another key component of program success, though participants also valued an open agenda that allowed them to discuss other important topics. Participants enjoyed learning from guest speakers on topics of interest, appreciating a reliable source amidst the exhaustive amount of information on the Internet. One participant said they would like a support group “that covers a lot of the topics that people would commonly look up and find a whole lot of different information from people who maybe should not be sharing that kind of information.” Other topics suggested by group members for future support groups are shown in Fig. 2.

Allowing open agenda time gave participants the space to discuss deeply personal and meaningful topics. For example, one participant shared that, in addition to “the common topics that new moms deal with as it relates to having a new baby, so sleep and breastfeeding,” it was also important to her to be “able to have open facilitated discussions surrounding the stuff people don’t want to talk about.” Another participant suggested the possibility of “spinoff groups” for “folks who are looking for more specialized engagement,” such as a fitness group or play date group.

### Accessibility is Key

Participants highlighted programming features that addressed barriers to participation in TNT-PISP and increased program accessibility. Key aspects contributing to its accessibility included being child-friendly and promoting flexible participation. Many participants relied on the ability to bring their children and receive a meal during group meetings. One participant expressed, “sometimes people don’t do things because of like, ‘ok my kids’ or like, ‘they haven’t had dinner’ or ‘I can’t get there’ like, there is no reason why they can’t come [to the support group] because you have made it all available to them.” Many participants were breastfeeding throughout their participation, making it essential for the program to support breastfeeding and pumping needs. One participant commented, “You’re still tied to that nursing baby…easier just to bring the baby with you and then you can leave the older child, you know, home with dad. That’s really helpful too. And sometimes it’s like this is a moms-only event and you’re kind of like, ‘Ugh then I have to pump.’ So, it’s really helpful that kids can come too.”

Participants appreciated the flexibility in the mode of participation (virtual or in-person) and the lack of an attendance policy. They found virtual meetings convenient; one participant shared, “I like the virtual format. It’s just super easy to hop on the phone. But if it were in-person, it would be harder for me, for sure, to get over—get in the car.”

During a virtual focus group meeting, a participant suggested a future hybrid option: “So I do see the importance of in-person, I do see the importance of virtual for those that can’t. There’s strength in both of those components, so maybe some kind of hybrid version of the two might be a better solution, so that those of us that can’t make it in person, we’ve got the virtual option and maybe participation rates will stay high or higher.”

As our participants were mothers with young children, they emphasized the importance of flexibility in attendance, as personal or familial needs sometimes affected their ability to attend the support group. One participant noted this struggle with a young infant, saying “there were just some nights where I’m like, you know, I’m really tryna get [baby’s name] down, I’m not gonna be able to sign in or just, you know, not feeling obligated but feeling like excited to go when I can.”

## Discussion

This study provides valuable insights for the design of programs that employ culturally informed and community-driven strategies to support Black women, as reflected in participants’ experiences with the TNT-PISP support groups. Participants identified several program elements as particularly meaningful, including dedicated time to discuss challenging topics, especially those specific to being a Black parent and coping with the effects of racism. Participants also valued the opportunity to problem-solve and address resource needs within the group. Furthermore, participants expressed appreciation for efforts to reduce systemic barriers to engagement, such as offering a child-friendly environment and holding sessions in a familiar, frequently visited community location.

Socioeconomic factors, medical racism, and mistrust of healthcare systems cause poor birth outcomes for Black women in Milwaukee (Capp, 2022; Zapata et al., 2025), has suggested group prenatal care as an opportunity to build trust and work towards health equity. Although results have been mixed on whether group prenatal care models, such as CenteringPregnancy, reduce disparities in preterm birth and low birth weight in Black infants, group prenatal care is positively viewed by participants (Catling et al., 2015; Chae et al., 2017; Crockett et al., 2022; Manant & Dodgson, 2011; McNeil et al., 2012). Group prenatal care has been shown to positively impact the psychosocial well-being of pregnant women suffering from high stress, which has been quantified using the Perceived Stress Scale in several studies (Heberlein et al., 2016; Ickovics et al., 2011). Several focus group studies relevant to our patient population have suggested that stress plays a role in birthing outcomes (McLemore et al., 2018; The Foundation for Black Women’s Wellness & EQT by Design, 2019). In addition to the benefits of engaging with a trusted care model, group care may also reduce psychosocial stress. Our study highlights the need for more research into culturally centered care models, as initial studies have shown that culturally centered birth centers (Hardeman et al., 2020; Liese et al., 2022), peer support groups in Black communities (Duncan et al., 2022; Shakya et al., 2017; Ware et al., 2021), and doula care can all promote health equity for Black women and their infants (Kozhimannil et al., 2013; Lemon et al., 2024; Ramey-Collier et al., 2023).

This study had several limitations. First, the study context and support group were tailored to meet the needs of a specific community; the experiences highlighted in these stories may differ from those in other contexts. Additionally, the transition to virtual meetings due to the COVID-19 pandemic may have influenced participants’ experiences and engagement. Future research should explore the long-term impacts of hybrid support models and address the scalability of such programs in different settings. As our study was qualitative, there are no outcome data to indicate whether our intervention reduces Black maternal and infant morbidity or mortality. However, when interpreted in the context of current literature, our data suggest that community-engaged, culturally relevant peer support has a role in addressing these disparities. Further quantitative research is needed to better characterize the effects of community-based and culturally relevant care models on Black maternal and infant morbidity and mortality.

## Conclusions for Practice

This study highlights the potential of community-based, culturally informed perinatal support programs to promote health equity for Black mothers and their infants. Future research should explore such programs’ long-term impacts and scalability in diverse settings. Continued efforts to integrate culturally relevant care models into traditional healthcare systems may help address health inequities in Black communities.

## Supplementary Information

Below is the link to the electronic supplementary material.

Supplementary Material 1 (DOCX 38 KB)

## Data Availability

Due to the identifying nature of interview transcripts, we have only provided the non-identifying quotes used in the manuscript.
