# Supplementary material for: Community-Led Solutions to Address Black Maternal and Infant Mortality Through the TNT-PISP Model: A Qualitative Study
Source: Matern Child Health J. 2026 May 2;30(5):673–80. doi: 10.1007/s10995-026-04264-1 (PMC13190512; doi:10.1007/s10995-026-04264-1)
Supplement: Supplementary file 1 — Supplementary Material 1 (DOCX 38 KB) [file 10995_2026_4264_MOESM1_ESM.docx]

Supplemental Materials:

**Community-Led Solutions to Address Black Maternal and Infant Mortality through the TNT-PISP Model: A Qualitative Study**

*Maternal and Child Health Journal*

**A. Composition and training of the research team:**

- One-on-one interviews were conducted by the principal investigator, JZ, who identifies as Black. A Black community-based doula also helped facilitate focus groups.
- A Black medical student (BFT) and a Black family medicine resident (MSW) performed coding of our initial paper under the supervision of a PhD researcher trained in qualitative methods (LETS).
- For this paper, a different Black medical student (DZ) and a Black family medicine attending (MSW, formerly a resident) re-coded the quotes related to the support group.
- JZ developed the interview guide in collaboration with community partners.
- All members of the research team identify as female. All team members except for LETS identify as Black.

**B. Participant and Interview Background**

- All participants identified as Black females as this was the recruitment criterion. Further demographic data was de-identified as part of the research protocol.
- Participants were recruited through word-of-mouth by community partners and flyers distributed around the community center and local clinics.
- Focus groups 1 and 2 each had 7 unique participants; focus group 3 consisted of participants who had already participated in either an individual interview or in the first two focus groups.
- Quotes from focus group 3 are not further identified.

**C. Qualitative Interview Guide**

Purpose: Thank you so much for taking the time to speak with me today. The reason I wanted to talk with you today is because my team is trying to learn more about how to best support pregnant Black women during their pregnancy and after childbirth. Currently, Black babies in Dane County die at 3 times the rate of white babies and this is a public health crisis we are taking very seriously. Your insights and experiences are extremely valuable to us so we can all work together to find solutions. By learning from you and other mothers, we hope to improve health care services given to pregnant Black women in Dane County and create new programs to address the needs of future mothers.

Confidentiality:

• There are no right or wrong answers.

• We just want to know what you think and your opinions.

• You will not be identified by name, and the information that you share with me will be kept confidential.

• Please be honest. You won’t hurt our feelings.

• We will be tape-recording this discussion and only project staff will hear these tapes.

• You can choose not to answer any question, and you can leave the study at any time.

• Your participation in this study will not affect the services that you receive from your provider.

• Information that we talk about today will not be given to your health care provider

**One-on-One Qualitative Interview 1/3 – During early pregnancy (First 6 months)** (Select questions from this question list will also be used for the community focus group)

1. I’d like to get to get to know you a little more, please tell me a little more about yourself. (Open ended question).
2. Congratulations on your pregnancy! Please tell me what you are most excited about and most worried about this pregnancy.
3. What stressors, if any, do you currently have in your life? What things do you currently do to combat your stress? What things do you wish you had that you don’t have now that would help you with these stressors?
4. Please tell me about what types of social support you have. Please elaborate.
5. What things bring you joy?
6. Have you ever been pregnant before? Can you please tell me a little bit about your prior pregnancies? What things did you like about your prior pregnancy? Anything you wish was different about your prior pregnancy?
7. In WI, Black women have higher rates of premature births and poor birth outcomes compared with white women. Why do you think that is? What things do you think could help improve that?
8. We are working to design a safe space for Black women to be supported during and immediately after their pregnancies, among other Black women, in a safe, supportive environment. We are currently designing this program and would love your input. What things would you like to see in a program like this? What things are important to you to create a safe, supportive atmosphere? What things do you think will draw people to a program like this and keep them coming?
9. Can you please discuss the value of having people on your birth support team (medical providers, doulas, nurses etc.) from the same racial/ethnic background as you? Is this something that is important to you? Why or why not? Please tell me your thoughts on this.
10. What types of topics do you think are most important to discuss in programs like the one we are designing?
11. If there were an opportunity to have onsite prenatal care (fundal measurements, one-on-one exam with a healthcare provider, opportunity to get individual questions answered) at the same community center where the support group meetings were held, would this be of value to you? Why or why not?
12. Where do you currently get your health information from? How do you learn best?
13. When you receive information from your healthcare provider, are you pleased with the way it is delivered? Why or why not? How can this be improved?
14. What are your thoughts on breastfeeding? Please tell me your exposure to breastfeeding growing up and currently.
15. If you plan to breastfeed, what are your current goals? What are perceived facilitators and barriers you anticipate in reaching your breastfeeding goals? What are your partners/friends/family’s attitudes toward breastfeeding?
16. Do you know what a doula is? Have you ever used a doula? If so, what have your experiences been?
17. What are your expectations from the healthcare system?
18. There are many Black women who distrust the healthcare system, can you please tell me your thoughts on this? Have you found this to be true? If so, why do you think this is the case? In your opinion, what are strategies to rebuild this trust?
19. Many Black women report stress in their lives from racism within society and in the healthcare system. Have you found this to be true? If so, please elaborate. How can Black women heal from this stress? In your opinion, what things need to be done in order to eliminate this racism and implicit bias within the healthcare system.
20. Do you feel supported as a parent? Are you aware of any parenting resources in the community? How confident are you in your ability to be a great parent?
21. Overall, what things do you feel you need to be supported in your pregnancy and beyond?
22. Are you interested in having more kids after this pregnancy? How do you plan to control the spacing of your pregnancies after this one?
23. Is there anything I didn’t ask about that you would like to say? Anything else on your mind?

Thank you so much for taking the time to meet with me today. I truly value and appreciate you. We will be using your input and comments to design and improve the way Black women receive prenatal care.

**One-on-One Qualitative Interview 2/3 – Early Postpartum** (0-5 months)

1. Congratulations on your new baby. Please tell me about your birthing experience. Was there anything that didn’t go as planned or that surprised you? What was your most positive memory? What do you love most about being a mom?
2. What stressors, if any, do you currently have in your life? What things do you currently do to combat your stress? What things do you wish you had that you don’t have now that would help you with these stressors?
3. Please tell me about what types of social support you have. Please elaborate. Has anything changed since the last time we talked?
4. In WI, Black women have higher rates of premature births and poor birth outcomes compared with white women. Why do you think that is? What things do you think could help improve that?
5. We are working to design a safe space for Black women to be supported during and immediately after their pregnancies among other Black women in a safe supportive environment. We are currently designing/improving this program and would love your continued input. What things would you like to see in a program like this? What things are important to you to create a safe, supportive atmosphere? (For participants in intervention group, will ask specifically what things they have enjoyed so far about the program and what things they’d suggest to take the program to the next level. Will ask them to discuss their overall experience in the program. Will specifically ask about the perceived value of group-based settings and community-based locations. Will ask what things drew them to the program and kept them coming back.)
6. Can you please discuss the value of having people on your birth support team (medical providers, doulas, nurses etc.) from the same racial/ethnic background as you. Is this something that is important to you? Why or why not? Please tell me your thoughts on this. Did you have any of these types of members throughout your pregnancy/birthing experience?
7. What types of topics do you think are most important to discuss in programs like the one we are designing? (For those who are participating in the TNT-PISP pilot program, will ask what topics have been helpful, which ones do they wish could be added)
8. If there were an opportunity to have onsite prenatal care (fundal measurements, one-on-one exam with healthcare provider, opportunity to get individual questions answered) at the same community center where the support group meetings were held would this be of value to you? Why or why not? If there were an opportunity to have on-site well-child exams, would this be beneficial?
9. Where do you currently get your health information from? How do you learn best?
10. When you receive information from your healthcare provider, are you pleased with the way it is delivered? Why or why not? How can this be improved?
11. Please discuss your decision-making process to breastfeed your baby or not. Please discuss your feeding/breastfeeding journey up until this point. What were perceived facilitators and barriers you anticipated in reaching your breastfeeding goals? Have those things come about to be true? What are your partners/friends/family’s attitudes toward breastfeeding, have they been supportive?
12. Do you know what a doula is? Have you ever used a doula? If so, what have your experiences been?
13. What are your expectations from the healthcare system?
14. There are many Black women who distrust the healthcare system, can you please tell me your thoughts on this? Have you found this to be true? If so, why do you think this is the case? In your opinion, what are strategies to rebuild this trust?
15. Many Black women report stress in their lives from racism within society and in the healthcare system. Have you found this to be true? If so, please elaborate. How can Black women heal from this stress? In your opinion, what things need to be done in order to eliminate this racism and implicit bias within the healthcare system?
16. Do you feel supported as a parent? Are you aware of any parenting resources in the community? How confident are you in your ability to be a great parent? (If participants are in TNT-PISP group, will ask if the program helped them increase their self-efficacy and perceived sense of support. Will ask them if they had prior pregnancies without participating in this support group how they compared to most recent pregnancy and being part of the TNT-PISP group. In what ways was the experience the same/different)
17. Overall, what things do you feel you need to be supported with this new baby?
18. Are you interested in having more kids after this pregnancy? How do you plan to control the spacing of your pregnancies after this one?
19. Is there anything I didn’t ask about that you would like to say? Anything else on your mind?

Thank you so much for taking the time to meet with me today. I truly value and appreciate you. We will be using your input and comments to design and improve the way Black women receive prenatal and postnatal/parenting support.

**One-on-One Qualitative Interview 3/3 – Late postpartum (6-12 months)** (Select questions from this question list will also be used for the TNT-PISP participant focus group)

1. Congratulations again on your new baby. How has everything been going? What new things is the baby doing? Any updates since the last time we met?
2. What stressors, if any, do you currently have in your life? What things do you currently do to combat your stress? What things do you wish you had that you don’t have now that would help you with these stressors?
3. Please tell me about what types of social support you have. Please elaborate. Has anything changed since the last time we talked?
4. In WI, Black women have higher rates of premature births and poor birth outcomes compared with white women. Why do you think that is? Based on your personal experiences, what things do you think could help improve that?
5. We are continually working to design a safe space for Black women to be supported during and immediately after their pregnancies among other Black women in a safe supportive environment. We are currently designing/improving this program and would love your continued input. What things would you like to see in a program like this? What things are important to you to create a safe, supportive atmosphere? (For participants in intervention group, will ask specifically what things they have enjoyed so far about the program and what things they’d suggest to take the program to the next level. Will ask them to discuss their overall experience in the program. Will specifically ask about -the perceived value of group based settings and community-based locations. Will also ask what things initially drew them to the program and kept them coming back. Will also ask their input on curriculum)
6. Reflecting back on your pregnancy and postpartum experience, can you please discuss the value of having people on your medical team (medical providers, doulas, nurses etc.) from the same racial/ethnic background as you. Is this something that is important to you? Why or why not? Please tell me your thoughts on this. Did you/do you have any of these types of members throughout your pregnancy/birthing experience? Do you currently have any now?
7. What types of topics do you think are most important to discuss in programs like the one we are designing? What topics are important to you now based on the current stage of parenting you are in? (For those who are participating in the TNT-PISP pilot program, will ask what topics have been helpful, which ones do they wish could be added)
8. If there were an opportunity to have onsite prenatal care (fundal measurements, one-on-one exam with healthcare provider, opportunity to get individual questions answered) at the same community center where the support group meetings were held would this be of value to you? Why or why not? If there were an opportunity to have onsite well child exams would this be beneficial?
9. Where do you currently get your health information from? How do you learn best?
10. When you receive information from your healthcare provider, are you pleased with the way it is delivered? Why or why not? How can this be improved?
11. Please discuss your decision-making process to breastfeed your baby or not. Please discuss your feeding/breastfeeding journey up until this point. What were the perceived facilitators and barriers you anticipated in reaching your breastfeeding goals? Have those things come about to be true? Did you reach your goals? What things helped or prevented you from reaching your breastfeeding goals, if any? What are your partners/friends/family’s attitudes toward breastfeeding? Have they been supportive?
12. Do you know what a doula is? Have you ever used a doula? If so, what have your experiences been?
13. What are your expectations from the healthcare system?
14. There are many Black women who distrust the healthcare system, can you please tell me your thoughts on this? Have you found this to be true? If so, why do you think this is the case? In your opinion, what are strategies to rebuild this trust?
15. Many Black women report stress in their lives from racism within society and in the healthcare system. Have you found this to be true? If so, please elaborate. How can Black women heal from this stress? In your opinion, what things need to be done in order to eliminate this racism and implicit bias within the healthcare system.
16. Do you feel supported as a parent? Are you aware of any parenting resources in the community? How confident are you in your ability to be a great parent? (If participants are in TNT-PISP group, will ask if the program helped them increase their self-efficacy and perceived sense of support. Will ask them if they had prior pregnancies without participating in this support group how they compared to most recent pregnancy and being part of the TNT-PISP group. In what ways was the experience the same/different)
17. Overall, what things do you feel you need to be supported with this new baby?
18. Are you interested in having more kids after this pregnancy? How do you plan to control the spacing of your pregnancies after this one?
19. Is there anything I didn’t ask about that you would like to say? Anything else on your mind?
20. What advice would you give to other Black mothers in the community about

Thank you so much for taking the time to meet with me today. I truly value and appreciate you. We will be using your input and comments to design and improve the way Black women receive prenatal and postnatal/parenting support.

**One-on-One Qualitative Interview with Key Collaborators involved in implementation of TNT-PISP Pilot (doula co-facilitators, academic partners, community-based partners, center staff, etc)**

1. What was your role in the implementation of the TNT-PISP?
2. What was your overall experience with participating in this launch?
3. In your opinion, what were facilitators and barriers to the implementation of this program? If any barriers, please discuss how they were overcome or how they can potentially be overcome?
4. Do you believe our team had the proper capacity to implement and maintain this program?
5. What was the most enjoyable thing about your participation in this project? Any special memories that stand out? What was the least enjoyable thing about your participation in this project?
6. Where do you see this pilot going from here? What things would you like to see happen as a result of the momentum we have created? What dreams do you have for this program?
7. Do you see any threats to the sustainability of this program? If so, what things can be done to overcome them?
8. If you could give advice for other sites interested in replicating what we have done, what would you tell them?

Thank you so much for your dedication and time being part of the implementation of the TNT-PISP. I truly value you and this information will be used for strategic planning moving forward and to help other site interested in starting a program like this in the future.

**Table 1: Themes with Illustrative Quotes**

| Themes | Illustrative quotes |
| --- | --- |
| Connection through shared identity and social support | “I definitely want to see Black people [at the support group]. Not saying that white people, white women wouldn't have an opinion or things like that, but I wouldn't get why they're really there unless they were a part of the study and just seeing how it compares. But in the support group, Black women [need to be there] so that we could be comfortable sharing what we want to share.”  *Participant 11, interview 1/1* |
|  | “And I think also that because every mother, every pregnancy is different, having access to so many Black women is essential because if anyone is gonna have a pregnancy similar to the one a mother is having…it would likely be a Black woman.”  *Participant 6, interview 3/3* |
|  | “Everyone got to tell their story. Everyone got to tell it with no judgment from anyone else.”  *Participant 1, interview 1/2* |
|  | “It was just really nice as- I’ve been using this term a lot- let your hair down kind of a thing. Whereby we can be our authentic selves because we don’t have enough spaces where we can be and to share. Even [though] what we say may not be the most correct, but things that are ailing us, bothering us, things we need clarity on.”  *Focus Group 3 participant* |
|  | “it helps to be surrounded by a community of Black women going through similar experiences to help you feel validated and to help you feel that you know I’m not just making things up or over exaggerating. These are truly terrible experiences that I’m going through and it’s normal to feel this way.”  *Participant 2, interview 1/2* |
|  | “When you’re surrounded by people who don’t have those experiences, it’s easy for you to just be like, ‘Oh no, I should just dismiss this’ or ‘Maybe it’s just me.’ To have that community validation and support- I think that is really important for improving mental health.”  *Participant 2, interview 1/2* |
|  | “And then also you're establishing community and also sustaining these moms with wonderful other women, with children, various ages support as they're navigating that first year or two of life or during mom with babe in utero.”  *Focus Group 3 participant* |
|  | “There's so much support in that group. And you feel it. Even though it's virtual, you feel it through the computer. You feel everyone caring about one another. And so, it's just really special because I don't feel-- I've lived in [city name] for a while. And I've never had that kind of experience with other Black women.”  *Participant 10, interview 3/3* |
| Community knowledge is valuable | “Yeah, I think for me just being able to learn like that from other moms and the wealth of knowledge shared and the different experiences and not…having to go talk to doctors or necessary medical people about just the day to day of being a mother and the different things that may come up that you can get answers from other mothers.”  *Focus Group 3 Participant* |
|  | “I think getting like-minded people together, or at least people who are experiencing the same thing, right? So pregnant women. Just to talk through what their successes and challenges are, what they're doing in their pregnancy, you know, how they plan to care for their children afterwards.”  *Participant 8, interview 1/1* |
|  | “I just shared a concern about how expensive daycare is and people came from out [of] the woodwork like, ‘Oh, get on this list,’ or ‘try this place,’ or ‘this place had really great reviews.’”  *Focus Group 3 Participant* |
|  | “So I teach on campus and then my partner is a student on campus and so [another participant] was just saying you know y’all absolutely have access to these funds to kind of help kind of decrease your contribution to daycare expenses. And so if I had not had that conversation, I wouldn’t have even known to look that up. And so it felt like there were questions that I could ask these folks but could not have asked my colleagues at work or my HR representative at work or that they might not have even known the answer to.”  *Participant 6, interview 3/3* |
| Agenda setting for and by participants | “And I think for me it would be like a [support group] space that covers a lot of the topics that people would commonly look up and find a whole lot of different information from people who maybe should not be sharing that kind of information.”  *Participant 10, interview 2/3* |
|  | “Yeah. I think it's like [what I’d like to hear at the support group] the common topics that new moms deal with as it relates to having a new baby, so sleep and breastfeeding but also being able to have open facilitated discussions surrounding the stuff people don't want to talk about.”  *Participant 10, interview 2/3* |
|  | So it was low pressure but high excitement when you did show. So I'm just wondering how there might be additional spinoff groups facilitated. So let's say somebody was looking for a mothers' group to work out with or looking for a mothers' group to just have some play dates or how this main group might be able to facilitate more focused or specialized groups for folks who are looking for more specialized engagement.”  *Focus Group 3 Participant* |
| Accessibility is key | “One thing that’s nice is that you are gonna—sometimes people don’t do things because of like “ok my kids” or like “they haven’t had dinner” or “I can’t get there” like there is no reason why they can’t come because you have made it all available to them [for the support group].”  *Focus Group 2, Participant 5 (of this focus group)* |
|  | “You’re still tied to that nursing baby…easier just to bring the baby with you and then you can leave the older child, you know, home with dad. That’s really helpful too. And sometimes it’s like this is a moms-only event and you’re kind of like, ‘Ugh then I have to pump.’ So, it’s really helpful that kids can come too.”  *Participant 5, interview 1/1* |
|  | “I like the virtual format. It’s just super easy to hop on the phone. But if it were in-person, it would be harder for me, for sure, to get over—get in the car.”  *Participant 10, interview 3/3* |
|  | “So I do see the importance of in-person, I do see the importance of virtual for those that can't. There's strength in both of those components, so maybe some kind of hybrid version of the two might be a better solution, so that those of us that can't make it in person, we've got the virtual option and maybe participation rates will stay high or higher.”  *Focus Group 3 Participant* |
|  | “…there were just some nights where I’m like, you know, I’m really tryna get [baby’s name] down, I’m not gonna be able to sign in or just, you know, not feeling obligated but feeling like excited to go when I can.”  *Participant 6, interview 3/3* |
